# Medial patellar ligament reconstruction in combination with derotational distal femoral osteotomy for treating recurrent patellar dislocation in the presence of increased femoral anteversion: a systematic review

**DOI:** 10.1186/s13018-024-04709-9

**Published:** 2024-04-06

**Authors:** Jinghong Yang, Jun Zhong, Han Li, Yimin Du, Xu Liu, Zhong Li, Yanshi Liu

**Affiliations:** 1 Department of Orthopedics, Affiliated Hospital of Southwest Medical University, Lu Zhou, 646000 China; 2 Department of Oncology, The Affiliated Hospital of Southwest Medical University, 25 TAIPING Street, Luzhou City, 646000 Sichuan Province China

**Keywords:** Medial patellar ligament reconstruction, Systematic review, Femoral anteversion, Derotational distal femoral osteotomy

## Abstract

**Background:**

Medial patellar ligament reconstruction (MPFL-R) in combination with derotational distal femoral osteotomy (DDFO) for treating recurrent patellar dislocation (RPD) in the presence of increased femoral anteversion is one of the most commonly used surgical techniques in the current clinical practice. However, there are limited studies on the clinical outcomes of MPFL-R in combination with DDFO to treat RPD in the presence of increased femoral anteversion.

**Purpose:**

To study the role of MPFL-R in combination with DDFO in the treatment of RPD in the presence of increased femoral anteversion.

**Methods:**

A systematic review was performed according to the PRISMA guidelines by searching the Medline, Embase, Web of Science, and Cochrane Library databases through June 1, 2023. Studies of patients who received MPFL-R in combination with DDFO after presenting with RPD and increased femoral anteversion were included. Methodological quality was assessed using the MINORS (Methodological Index for Nonrandomized Studies) score. Each study’s basic characteristics, including characteristic information, radiological parameters, surgical techniques, patient-reported outcomes, and complications, were recorded and analyzed.

**Results:**

A total of 6 studies with 231 patients (236 knees) were included. Sample sizes ranged from 12 to 162 patients, and the majority of the patients were female (range, 67-100%). The mean age and follow-up ranges were 18 to 24 years and 16 to 49 months, respectively. The mean femoral anteversion decreased significantly from 34° preoperatively to 12° postoperatively. In studies reporting preoperative and postoperative outcomes, significant improvements were found in the Lysholm score, Kujala score, International Knee Documentation Committee score, and visual analog scale for pain. Postoperative complications were reported in all studies, with an overall reported complication rate of 4.7%, but no redislocations occurred during the follow-up period.

**Conclusion:**

For RPD with increased femoral anteversion, MPFL-R in combination with DDFO leads to a good clinical outcome and a low redislocation rate. However, there was no consensus among researchers on the indications for MPFL-R combined with DDFO in the treatment of RPD.

## Introduction

Recurrent patellar dislocation (RPD) is a frequent knee condition in young individuals and is second only to anterior cruciate ligament rupture in terms of the frequency of knee injuries [1–4]. Medial patellofemoral ligament reconstruction (MPFL-R) is currently considered an effective treatment for RPD [5–9]. However, there are many different risk factors for RPD [10], and in many cases, medial patellar ligament reconstruction (MPFL-R) alone may not address the different risk factors for RPD. Instead, a combination of surgical procedures may be the best course of action [1].

There are several risk factors for RPD, and an increased femoral anteversion angle (FAA) has been suggested as one of the risk factors affecting RPD [11–15]. The FAA was defined as the angle measured in the horizontal (or transverse) plane between the knee joint axis and the hip joint axis, indicating the degree of torsion of the femur, which can be measured by CT or MRI. Recent biomechanical studies have suggested that an increased FAA may significantly increase the lateral stresses acting on the patella and may be a cause of MPFL-R failure [16, 17]. To date, there is still some controversy and discussion regarding the treatment of patients with FAA-increased patellar dislocation. Some studies suggest that MPFL-R alone is sufficient to compensate for bony structural abnormalities [18–20], while most studies have suggested that the addition of DDFO is beneficial in patients with FAA-increased RPD [21–25]. Although there is a systematic review of DDFO for treating RPD in the presence of increased FAA [26], studies of MPFL-R combined with DDFO for treating FAA-increased RPD are limited to small case series, and no study has attempted to aggregate their data.

The purpose of this study was to investigate the role of medial patellofemoral ligament reconstruction combined with DDFO in treating RPD in the presence of increased femoral anteversion. It was hypothesized that favorable functional outcomes and low redislocation rates would be found after medial patellofemoral ligament reconstruction combined with DDFO.

## Methods

### Literature research

Firstly, we registered for the systematic review on the Prospero website (https://www.crd.york.ac.uk/PROSPERO). Then, a systematic review was performed following the PRISMA guidelines (Preferred Reporting Items for Systematic Reviews and Meta-analyses) [27–33]. The Medline, Embase, Web of Science, and Cochrane Library databases were searched by all of the authors independently on June 1, 2023. The search terms were as follows: (patellar dislocation OR patellar instability OR patellar subluxation OR patellofemoral dislocation OR patellofemoral dysfunction) AND (rotational osteotomy OR derotational osteotomy OR torsional osteotomy) AND (MPFL reconstruction OR medial patellofemoral ligament reconstruction OR MPFL OR medial quadriceps tendon femoral ligament OR medial quadriceps tendon femoral ligament OR MQTFL OR medial patellofemoral complex).

### Study selection

All of the publications found by these search terms were reviewed, and the inclusion and exclusion criteria were then negotiated among the authors. The senior author (Z.L. or Y.L.) made the final determination regarding whether a study should be included if there were any disagreements among the authors. Then, full-text publications were reviewed critically. In addition, all references in the study were reviewed and checked to verify that no relevant articles had been omitted from the systematic review. All studies were included that reported the clinical outcomes of MPFL-R in combination with DDFO for FAA-increased RPD. The main purpose of this systematic review was to investigate the role of MPFL-R in combination with DDFO for treating RPD in the presence of increased femoral anteversion; therefore, patients with a history of undergoing ipsilateral derotational tibial osteotomy were excluded, and patients with other combined procedures were excluded. The detailed inclusion and exclusion criteria are described in Table 1.

**Table 1:** Inclusion and exclusion criteria of the systematic review

Inclusion Criteria	Exclusion Criteria
Patients with recurrent patellar dislocation	History of ipsilateral tibial osteotomy Cadaveric, biomechanical, or animal study
A minimum follow-up of 12 months	
History of derotational femoral osteotomy or medial patellar ligament reconstruction	Systematic review or meta-analysis Patient duplication
Evidence levels 1–4	Surgical technique article or case report

### Data extraction

Two reviewers (J.Y. and J.Z.) independently extracted the data. Regarding research features, patient characteristics, surgical procedures, and outcome measures, an abstract of each full-text article was created. Means and standard deviations were extracted from the data on pre- and postoperative outcome assessments. The von Elm et al. [34] decision tree was used to find and omit duplicate research. Any discrepancies were resolved through discussion with a third author (Z.L.).

Study characteristics included publication date, study design, level of evidence, number of patients/knees, and length of follow-up. Characteristic data consisted of patient sex and age.

Outcome measures consisted of pre-and postoperative clinical and radiographic evaluations. Clinical assessments included patient-reported function scores and objective patellar stability (MPFL residual laxity). Radiographic measurements included the pre-and postoperative FAA and patellar tilt angle.

### Risk of bias assessment

The methodological quality of each study was assessed independently by two review authors (J.Y. and J.Z.) according to the MINORS (Methodological Index for Nonrandomized Studies) score [35], The items are scored 0 (not reported), 1 (reported but inadequate) or 1 (reported and adequate). The maximum possible score is 16 for noncomparative studies and 24 for comparative studies. Low risk of bias was defined as MINORS scores of 13 to 16 for noncomparative studies and 21 to 23 for comparison studies, whereas high risk was defined as MINORS scores of ≤ 12 and ≤ 20 for noncomparative studies and comparative studies, respectively.

### Data analysis

Heterogeneity of individual studies on patient groups and treatments hindered meta-analysis calculations. Therefore, descriptive statistics were used to analyze age data and follow-up data as well as clinical and radiological results. The Cohen ĸ coefficient was used to calculate reliability statistics to measure the level of agreement among the raters for the MINORS scores. When the standard deviation was not available, the authors were contacted to provide the missing data, or it was calculated according to the Cochrane handbook.

## Results

### Study identification

**Fig. 1:**
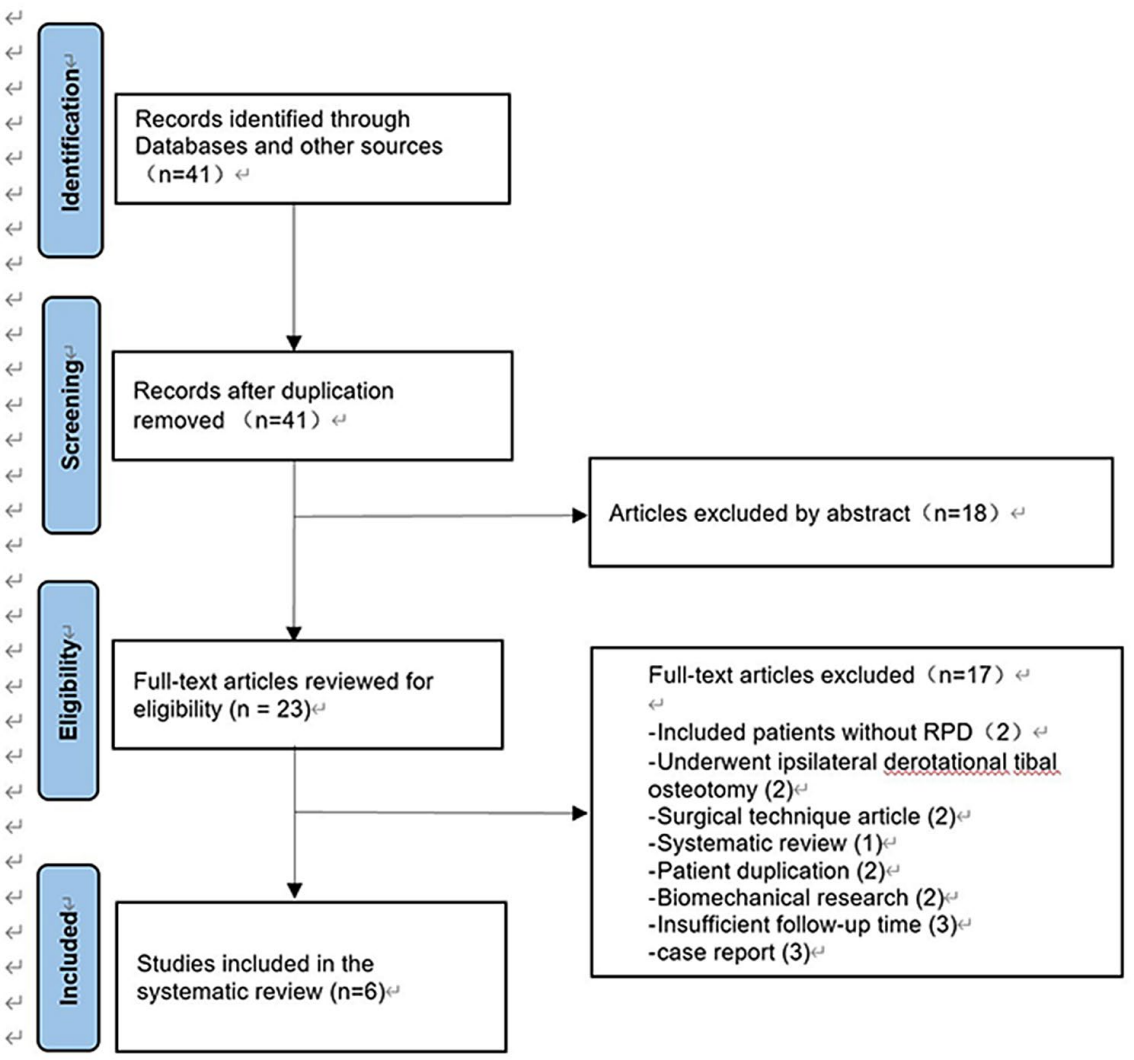
Flowchart of the search strategy following the PRISMA guidelines. RPD, recurrent patellar dislocation

Firstly, we registered our systematic review with ID CRD42023449720 on the Prospero website (https://www.crd.york.ac.uk/PROSPERO). Of the 41 studies identified, 34 were reviewed in full text. Ultimately, 6 studies were included in the systematic review (Fig. 1). 4 studies were retrospective case series (level 4) [23, 36–38], and 2 studies were retrospective cohort studies (level 3) [39, 40]. The mean MINORS score was 12.0 (range, 11–13) for the 4 noncomparative studies, and the two comparative studies’ mean score was 19.5 (Table 2) [39, 40]. Of the 6 studies, 4 were assessed as having a high risk of bias (MINORS score < 13 for a noncomparative study or < 21 for a comparative study) [23, 36, 38, 39].

**Table 2:** Study characteristics^a^

Lead Author	Year	Study Design	LOE	MINORS Score	Patient(knees)No.	Mean Age, y	Male/Female No.	Mean Follow-up, mo
Zhang	2021	Cohort study^b^	3	20/24	66(66)	21	59/7	37
Hao	2022	Cohort study^b^	3	19/24	31(31)	24	4/27	43
Deng	2021	Case series	4	12/16	13(13)	19	4/9	27
Zhang	2023	Case series^c^	4	13/16	92(102)	21	8/94	49
Nelitz	2015	Case series	4	12/16	12(12)	18	0/12	16
Li	2021	Case series	4	12/16	17(17)	18	5/12	18

^a^LOE, level of evidence; MINORS, Methodological Index for Nonrandomized Studies;

^b^This study compared the results of patients undergoing derotational femoral osteotomy vs. a nonderotational osteotomy procedure

^c^This study reported clinical results after combined derotational distal femoral osteotomy, which included 102 derotational femoral osteotomies, and an additional tibial tubercle osteotomy was performed in 26 knees. All patients who underwent derotational distal femoral osteotomy were included in the systematic review

### Patient characteristics

There were 231 patients (231 knees) in the 6 studies. Each study is summarized in Table 2. Age was cited in all 6 studies, with the mean ranging from 18 to 24 years. Sex distribution was 161 women (67%) and 80 men (33%) in 6 studies [23, 36–39]. All studies noted the follow-up intervals, resulting in a mean follow-up from 16 to 49 months.

### Surgical techniques

Table 3 depicts the surgical details across the studies. All studies reported the cut-off value of femoral anteversion for DDFO (range, 25°-30°) and all studies combined MPFL-R and DDFO, The preoperative mean FAA ranged from 33° to 35° (mean, 34°) in all studies, and the postoperative mean FAA ranged from 10° to 19° (mean, 14°) in 5 studies [36–39]. Supracondylar derotational osteotomy was performed and the pedicled quadriceps or semitendinosus tendon was used to rebuild the MPFL in all studies. Of the 6 studies, 2 reported that additional bony procedures were performed to correct the concurrent bony deformities [37, 39], such as tibial tubercle transfer, tibial tubercle medialization (TTM), and tibial tubercle distalization (TTD). However, our study only included patients who underwent MPFLR in combination with DDFO, as the inclusion of other procedures may have affected the outcome.

**Table 3:** Operative procedure data^a^

Lead Author	Cut-off Value for Osteotomy	Mean Pre/Post FAA (Modality)	Combined Bony Procedures
Zhang	30	34°/10° (3D CT)	TT transfer (*n* = 30)
Hao	25	33°/13°(axial CT)	none
Deng	25	33°/19° (axial CT)	none
Zhang	30	35°/11°(axial CT)	TTM(*n* = 18), TTD*n* = 5, TTM + TTD*n* = 3
Nelitz	25	34°/NR (axial MRI)	None
Li	30	35°/16°(axial CT)	None

aOsteotomy level for each study: distal femur. 3D, 3-dimensional; CT, computed tomography; FAA, femoral anteversion angle; MPFL-R, medial patellofemoral ligament reconstruction; MRI, magnetic resonance imaging; NR, not reported; Pre/Post, pre- and postoperative; TT, tibial tubercle; TTM, tibial tubercle medialization; TTD, tibial tubercle distalization

**Subjective Clinical Outcomes**.

The articles described 6 patient-reported outcomes. The VAS score for pain, IKDC score, Tegner score, Kujala score, and Lysholm score were used as outcome measures in ≥ 3 studies and are summarized in Table 4. All studies have shown significant improvement from preoperative to postoperative.

**Table 4:** Functional scores^a^

Lead Author	Tegner Score		Kujala Score		Lysholm Score		VAS Score		IKDC Score	
	Preop	Postop	Preop	Postop	Preop	Postop	Preop	Postop	Preop	Postop
Zhang^b^	3.2 ± 0.6	4.4 ± 0.8	53.8 ± 11.2	82.3 ± 8.4	58.2 ± 10.2	83.7 ± 9.0			56.7 ± 11.2	83.1 ± 10.4
Hao			54.5 ± 9.7	85.1 ± 7.7	56.6 ± 9.6	86.8 ± 8.2			53.8 ± 9.3	86.2 ± 10.0
Deng	2.2 ± 1.3	4.5 ± 1.8	57.48 ± 8.76	87.43 ± 4.25	59.9 ± 9.7	83.9 ± 6.5	4.8 ± 2.1	1.8 ± 1.5	51.4 ± 8.4	83.6 ± 7.3
Zhang	3.2 ± 0.6	4.6 ± 0.7	55.3 ± 8.4	84.5 ± 9.3	57.3 ± 7.4	85.5 ± 10.5	4.5 ± 0.8	1.5 ± 0.4		
Nelitz	4	4.5	69	92.5			4	1.5	60	85
Li	3.6 ± 1.1	6.8 ± 1.5	56.8 ± 5.7	89.0 ± 8.0	47.8 ± 8.1	93.4 ± 7.8	4.2 ± 1.1	2.4 ± 1.4		

^a^Blank cells indicate not reported. Values are presented as the mean ± SD. IKDC, International Knee Documentation Committee; Postop, postoperative; Preop, preoperative; VAS, visual analog scale

^b^The postoperative functional scores were significantly higher in the derotational group than in the control group

Only 2 studies reported patient satisfaction [23, 40], with a postoperative satisfaction rate ranging from 92 to 96%. A study compared the clinical outcome of treating RPD with FAA > 30° with MPFL-R and without DDFO [39], which showed that the former was more capable of yielding good subjective results than MPFL-R alone, and this result was even more striking in patients who had high-grade J-signs before surgery.

### Radiological and objective clinical outcomes

Postoperative osteotomy that did not heal was reported in 1 study and this patient had a satisfactory outcome after a second surgical revision to strengthen the fixation [38]. The limited knee flexion after they had been reported in the other 3 papers, fortunately, all 6 patients ended up with good clinical outcomes. One study found by a second arthroscopy that the patellofemoral articular cartilage lesions remained unchanged in most cases after the combined use of MPFL-R and DDFO [37]. Moreover, high-grade trochlear dysplasia and arthroscopic residual patellar maltracking might be associated with cartilaginous deterioration at the medial patellar facet after surgery.

### Complications

Five studies reported postoperative complications (Table 5). A total of 11 complications were noted for a rate of 4.5%. The most common complication was limited knee flexion (2.4%). All the patients resolved the limited knee flexion by enhanced rehabilitation, no patients experienced patellar subluxation or re-dislocation postoperatively. Only one patient had a post-operative osteotomy that did not heal.

**Table 5:** Complication, satisfaction rate, and radiological assessments^a^

Lead Author	Complications	Satisfaction, %^b^	Other Assessments
Zhang	MPFL residual graft laxity(*n* = 4)	Not reported	Residual J-sign, patellar medial laxity index
Hao	Limited knee flexion (*n* = 1)	96/4/0	Apprehension sign, J-sign, BPII score
Deng	none	Not reported	None
Zhang	Limited knee flexion (*n* = 3)	Not reported	J-sign, Patellar tilt
Nelitz	Limited knee flexion (*n* = 2)	92/8/0	None
Li	Osteotomy does not heal(*n* = 1)	Not reported	none

^a^No patients experienced patellar redislocation; however, 2.4% of patients after derotational femoral osteotomy showed medial patellofemoral ligament residual graft laxity

^b^Satisfied/partially satisfied/dissatisfied

## Discussion

The most important finding of this systematic review is that in the treatment of patients with RPD with increased FAA, DDFO combined with MPFL-R leads to good subjective and objective outcomes and arthroscopic review results, and patients have a low rate of postoperative re-dislocation, fewer postoperative complications, and higher patient satisfaction. Currently, there is a lack of consensus among researchers regarding the indications for the use of DDFO in combination with MPFL-R in the treatment of RPD. However, the present study provides evidence that the combination of MPFL-R and DDFO may lead to a good clinical outcome and lower rates of re-dislocation in the treatment of RPD patients with elevated FAA levels.

MPFL-R, a first-line surgical treatment option for patients with RPD, has excellent results for patients with RPD. However, RPD is influenced not only by the MPFL but also by the soft tissues and bony structures surrounding the knee joint, Yang et al. compared the correlation between femoral torsion and distal femoral morphology by imaging and discovered that an increased FAA often coincided with femoral trochlear dysplasia and that an increased FAA also had an impact on TT-TG and thus on patellofemoral stability [41]. Some researchers believe that the reconstruction of the MPFL alone is enough to treat patients with an increased FAA. Erickson et al. [19] declared that patients after MPFL-R got significantly better postoperative outcome scores and lower re-dislocation (1.1%), regardless of whether the FAA was high or low. Concurrently, orthopedic surgeons have favored MPFL-R over DDFO due to its relatively less invasive nature. However, some argue that for RPD patients exhibiting elevated FAA levels, the combination of MPFL-R and DDFO is preferable to correct femoral rotation intraoperatively, thereby achieving superior clinical outcomes. Some biomechanical studies have shown that MPFL-R alone is not enough to correct the increase of FAA in patients, Kaiser’s study confirmed that from a biomechanical point of view, MPFL-R alone for patellar instability seems to be reasonable for FAA to increase RPD less than 10 °, but for patients whose FAA > 20°, it is not enough to treat a higher degree of femoral torsion. Cao et al. [42] considered that MPFL-R revision by DDFO combined with MPFL-R achieved favorable clinical outcomes in patients with increased femoral anteversion along with a high-grade J sign. Franciozi [43] discovered that FAA > 30° has a negative effect on MPFL-R combined with tibial tubercle osteotomy (TTO) in the treatment of RPD. This coincides with the research results of Zhang et al. Therefore, for patients with FAA > 30°, MPFL-R combined with DDFO may be the best surgical method.

However, the FAA threshold for DDFO has not yet been determined, some scholars believe that FAA > 25° is the sign of starting DDFO, and MPFL-R combined with DDFO surgery has been performed on these patients, and good clinical outcomes have been achieved [23, 36, 40]. The study by Zhang et al. [39] indicated that the efficacy of MPFL-R combined with DDFO was also satisfactory at FAA > 30°, and they found that when preoperative patients presented with a J-sign, patients with a preoperative high-grade J-sign had inferior clinical outcomes, more MPFL residual graft laxity, and greater residual patellar maltracking. In the treatment algorithm for RPD proposed by Frosch and Schmeling [44] isolated MPFL-R was advocated when patellar tracking was normal, regardless of the increased FAA, and DDFO was to be performed only in the presence of patellar maltracking. Zhang et al. [37] found in another secondary arthroscopic observation study of 102 patients who after isolated DDFO, patellar tracking improved in the majority of cases. Immediately after MPFL-R, all preoperative patellar maltracking was reduced for all knee flexion angles.

Therefore, there was no consensus among researchers on the indications for MPFL-R combined with DDFO in the treatment of RPD. The study on indications for MPFL-R combined with DDFO in the treatment of RPD is currently lacking in depth. It still needs to be explored further.

Imaging studies have shown that other bone deformities may exist in patients with an increased FAA at the same time. How to treat these concurrent bony deformities is still debated in the literature. The patients in our study were also often accompanied by abnormalities such as increased TT-TG and trochlear dysplasia, Other bony deformities may coexist in patients with an increased FAA. How to treat these concurrent bony deformities is still debated in the literature. For the increase of TT-TG, some scholars believe that the increase of TT-TG is related to the increase of FAA, and correction of FAA is sufficient to correct the increase of TT-TG, while some scholars believe that TTO can be added for patients with excessive TT-TG (TT-TG > 20 mm), and some scholars believe that MPFL-R is strong enough to compensate for the increase of TT-TG and FAA, so TTO is generally unnecessary, and only some patients with excessive TT-TG can be considered for treatment with MPFL-R combined with TTO. A recent meta-analysis proposed that for patients with trochlear dysplasia, combined trochleoplasty reduces the rate of re-dislocation, but it will lead to an increased risk of postoperative functional limitations. While the latest retrospective analysis by Zhou et al. [45] showed that MPFL-R combined with DDFO showed satisfactory clinical outcomes during follow-up in patients with RPD who had excessive femoral anteversion angles and trochlear dysplasia. Even patients with high-grade trochlear dysplasia showed satisfactory results. Therefore, based on Zhou’s article, we think that for RPD patients with an increased FAA, trochleoplasty may be generally not recommended.

The study has several limitations. The low levels of evidence, heterogeneity bias, and retrospective nature of most studies are the major limitations of this systematic review. These limitations are common in systematic reviews of orthopaedic surgical procedures and are mostly unavoidable until higher levels of evidence-level studies on the topic are conducted and published.

## Conclusion

For RPD with increased anterior femoral anteversion, MPFL-R in combination with DDFO leads to a good clinical outcome. However, there was no consensus among researchers on the indications for MPFL-R combined with DDFO in the treatment of RPD.

## Data Availability

No datasets were generated or analysed during the current study.

## Abbreviations

RPD: Recurrent Patellar Dislocation
MPFL: Medial Patellar Ligament
DDFO: Derotational Distal Femoral Osteotomy
FAA: Femoral Anteversion Angle
MINORS: Methodological Index for Nonrandomized Studies
